# Interrogating bromodomain inhibitor resistance in KMT2A-rearranged leukemia through combinatorial CRISPR screens

**DOI:** 10.1073/pnas.2220134120

**Published:** 2023-04-10

**Authors:** Shaela Wright, Jianzhong Hu, Hong Wang, Judith Hyle, Yang Zhang, Guoqing Du, Marina Y. Konopleva, Steven M. Kornblau, Mohamed Nadhir Djekidel, Wojciech Rosikiewicz, Beisi Xu, Rui Lu, Jun J. Yang, Chunliang Li

**Affiliations:** ^a^Department of Tumor Cell Biology, St. Jude Children’s Research Hospital, Memphis, TN 38105; ^b^Department of Pharmaceutical Sciences, St. Jude Children’s Research Hospital, Memphis, TN 38105; ^c^Center for Proteomics and Metabolomics, St. Jude Children’s Research Hospital, Memphis, TN 38105; ^d^Department of Leukemia, The University of Texas MD Anderson Cancer Center, Houston, TX 77030; ^e^Center for Applied Bioinformatics, St. Jude Children’s Research Hospital, Memphis, TN 38105; ^f^Division of Hematology/Oncology, O’Neal Comprehensive Cancer Center, University of Alabama at Birmingham, Birmingham, AL 35294; ^g^Department of Oncology, St. Jude Children’s Research Hospital, Memphis, TN 38105

**Keywords:** genome editing, bromodomain inhibitor, KMT2A-rearranged leukemia, SPOP

## Abstract

Bromo- and extra-terminal domain inhibitors (BETi) have exhibited therapeutic activities in many cancers. However, the mechanisms controlling BETi response and resistance are not well understood. We conducted genome-wide loss-of-function CRISPR screens using BETi-treated KMT2A-rearranged (KMT2A-r) cell lines. We revealed that *Speckle-type POZ protein (SPOP)* gene (Speckle Type BTB/POZ Protein) deficiency caused significant BETi resistance, which was further validated in cell lines and xenograft models. Proteomics analysis and a kinase-vulnerability CRISPR screen indicated that cells treated with BETi are sensitive to GSK3 perturbation. Pharmaceutical inhibition of GSK3 reversed the BETi-resistance phenotype. Based on this observation, a combination therapy regimen inhibiting both BET and GSK3 was developed to impede KMT2A-r leukemia progression in patient-derived xenografts in vivo. Our results revealed molecular mechanisms underlying BETi resistance and a promising combination treatment regimen of ABBV-744 and CHIR-98014 by utilizing unique ex vivo and in vivo KMT2A-r PDX models.

## Significance

Using combinatorial loss-of-function CRISPR screens, our study revealed a Bromo- and extra-terminal domain inhibitors (BETi) resistance network in KMT2A-r leukemia. Based on these findings, we have also revealed a corresponding synergy treatment regimen, significantly improving strategies to prevent and treat BETi resistance and relapse that might be seen in the clinic.

Mix lineage leukemia (MLL)/KMT2A-rearranged leukemia represents one of the most refractory acute leukemia subtypes. It comprises ~10% of human leukemias, including more than 80% of infant cases, and manifests as ~5 to 10% of acute lymphoblastic leukemia (ALL) or acute myeloid leukemia (AML) (1–3). Over the past decades, improved understanding of diagnosis, multiagent combination chemotherapy, and supportive care have significantly increased the cure rate of ALL. However, patients with KMT2A-r ALL still face a dismal prognosis, and few therapeutic options are available for these patients (3–6). Recently, the CD19 CAR T-cell therapy of KMT2A-r B-ALL was reported to induce lineage-switch events and mutate surface antigens as the dominant relapse mechanisms (7–10). Nevertheless, the signaling circuitry and genomic abnormalities in KMT2A-r leukemia pathogenesis are complex, and the oncogenic drivers and genetic dependencies remain poorly characterized.

Genomic sequencing of ALL patients revealed very few genetic mutations besides the KMT2A translocations for this cancer. KMT2A-r pathogenesis most frequently results from a translocation between the N terminus of the *KMT2A* gene and a partner gene leading to an oncogenic chimeric protein. So far, more than 100 partner proteins have been identified, with most belonging to the super elongation complex family (5). These observations suggest that epigenetic and transcriptional dysregulation is essential in KMT2A-r leukemogenesis and tumor maintenance. Therapeutic targeting of the fusion proteins and their recruited/interacted proteins, including DOT1L, MEN1, LEDGF, and BRD4, has been attempted with mixed success. For instance, the well-characterized histone methyltransferase DOT1L is an upstream epigenetic regulator of *HOXA9.* Through its recruitment to the *HOXA* locus via KMT2A-r fusion proteins, DOT1L causes hypermethylation of the lysine 79 residue of histone H3 (11), and selective DOT1L inhibitors prevent the development of KMT2A-r leukemias (12, 13). However, these agents usually act slowly and exert suboptimal effects in clinical trials, limiting their further application. Therefore, more effective therapeutic strategies for patients with KMT2A-r leukemias are urgently needed.

Bromodomains (BDs) contain approximately 110 amino acids that recognize acetylated lysine in histones. BD-containing proteins play essential roles in chromatin remodeling and epigenetic regulation, influencing the transcription of many oncogenes, especially *MYC*, *BCL2*, *FLT3*, *NPM1*, *TP53*, and *Cyclin D1* (14–19). The well-established therapeutic target BRD4 is a member of the bromo- and extra-terminal domain (BET) family, which activates *MYC*’s enhancers, thereby maintaining its high oncogenic overexpression in KMT2A-r leukemia and other cancers. Several groups successfully developed multiple small-molecule drugs, such as JQ1 and I-BET762, to target the BET BDs and achieved encouraging potency in vitro and in vivo (19, 20). The second-generation small molecules ABBV-744 and iBET-BD2, which selectively target BD2 on BET proteins, have recently been developed (14, 21). Compared with the first-generation dual BET inhibitors that block BD1 and BD2, ABBV-744 exhibits greater potency and less toxicity (14, 21). Although BETis hold great promise for improving therapeutic potential, acquired BETi resistance was observed in PRC2-deficient and WNT-activated AML cells upon JQ1 treatment (22, 23). The molecular mechanisms governing drug response and resistance of these BETis remains poorly understood.

In this study, we focused on developing alternative therapeutic approaches to overcome acquired BETi resistance by exploring the molecular pathways uniquely activated in BETi-resistant leukemia cells. By conducting drug treatment–based combinatorial functional CRISPR screens, comprehensive validation with cell lines and patient-derived xenografts (PDXs), and mechanistic studies, we identified the *SPOP* as a critical gene responsible for BETi resistance in KMT2A-r leukemia. Furthermore, targeting the GSK3 pathway efficiently overcame BETi resistance. Inhibition of BETs and GSK synergistically inhibited the progression of KMT2A-r PDXs in vivo. In summary, our study elucidated the dysregulated BETi-resistance network in KMT2A-r leukemia and identified potential strategies to overcome BETi resistance.

## Results

### Combinatorial CRISPR Screens Identified *SPOP* as the Top Driver for BETi Resistance in the KMT2A-r AML Cell Line OCI-AML2.

**Fig. 1.**
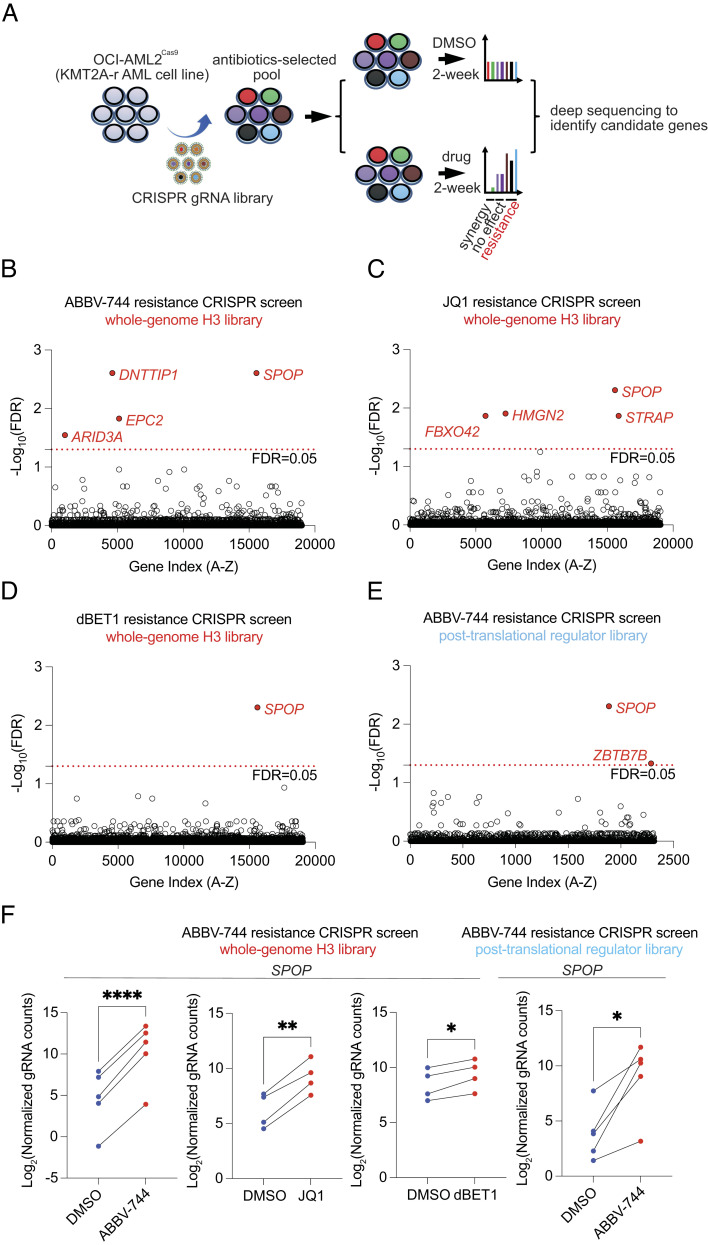
CRISPR screens identified *SPOP* as the top candidate gene responsible for BETi resistance. (*A*) Schematic diagram of a loss-of-function CRISPR screen to identify candidate genes that mediate BETi resistance by using KMT2A-r AML OCI-AML2 cells. (*B*–*E*) The enrichment score of several sgRNAs against each gene was combined by the MAGeCK algorithm. Top resistance-associated candidate genes were highlighted based on Log_10_(FDR). The whole-genome H3 library was combined with ABBV-744 in *B*, JQ1 in *C*, and dBET1 in *D*. The posttranslational regulator library was combined with ABBV-744 in *E*. (*F*) The overall distribution of all sgRNAs against *SPOP* is shown based on normalized gRNA counts, which were collected from MAGeCK analysis. Paired *t* test *P < 0.05, **P < 0.01, ***P < 0.001, ****P < 0.0001.

CRISPR-mediated loss-of-function screen is a powerful tool for identifying candidates associated with many biological phenotypes in an unbiased manner (24–26). Many CRISPR screens were performed in cancer models and identified candidate genes responsible for BETi resistance (26–28). To interrogate the BETi-resistance mechanism in leukemia models, we carried out an unbiased screen using the OCI-AML2 cell line (carrying the KMT2A-AF6 fusion) stably expressing Cas9 and a whole-genome CRISPR sgRNA library (H3, Addgene #133914) targeting more than 19,000 human genes. We reasoned that when genes targeted with CRISPR confer resistance upon drug treatment, cells carrying the corresponding sgRNA would be enriched and identified by deep sequencing. To this end, the Cas9 and CRISPR sgRNA library-targeted pools (M.O.I. = 0.3) of OCI-AML2 cells were selected by antibiotics (blasticidin for Cas9 and puromycin for the sgRNA library) to enrich for targeted cells, followed by a 2-wk continuous in vitro culture with ABBV-744 (BD2-specific inhibitor, 100 nM), JQ1 (dual-BD inhibitor, 100 nM), dBET1 (BRD4 PROTAC, 10 nM), or dimethyl sulfoxide (DMSO) as a negative control. Each cell population was split and supplemented with fresh drugs every 3 to 4 d. On day 14, cells were collected and subjected to genomic extraction, PCR, and the next-generation sequencing, followed by sgRNA deconvolution, and sgRNA-enrichment analysis using the MAGeCK algorithm (Fig. 1*A*) (29). Two biological replicate screens were conducted for each library, which demonstrated high consistency between replicates (*SI Appendix*, Fig. S1). Notably, with a stringent cutoff of FDR less than 0.05, SPOP was identified as the top hit in JQ1-, ABBV-744-, and dBET1-mediated whole-genome CRISPR screens (Fig. 1 *B*–*D*). An additional screen targeting posttranslational regulators (a customized CRISPR library containing ~10,000 gRNAs against ~1,500 genes associated with ubiquitination or deubiquitination) was used in OCI-AML2 Cas9-expressing cells as described above with continuous treatment of 100 nM ABBV-744 for 2 wk (Fig. 1*E*). In both the whole-genome and posttranslational regulator screens, 4 to 5 sgRNAs against independent exons of *SPOP* were significantly enriched (Fig. 1*F*). In summary, our combinatorial CRISPR screen identified SPOP as the top BETi resistance–associated gene in vitro.

### Validation of BETi-Resistance Phenotype Associated with SPOP Deficiency in Leukemia Cell Lines.

**Fig. 2.**
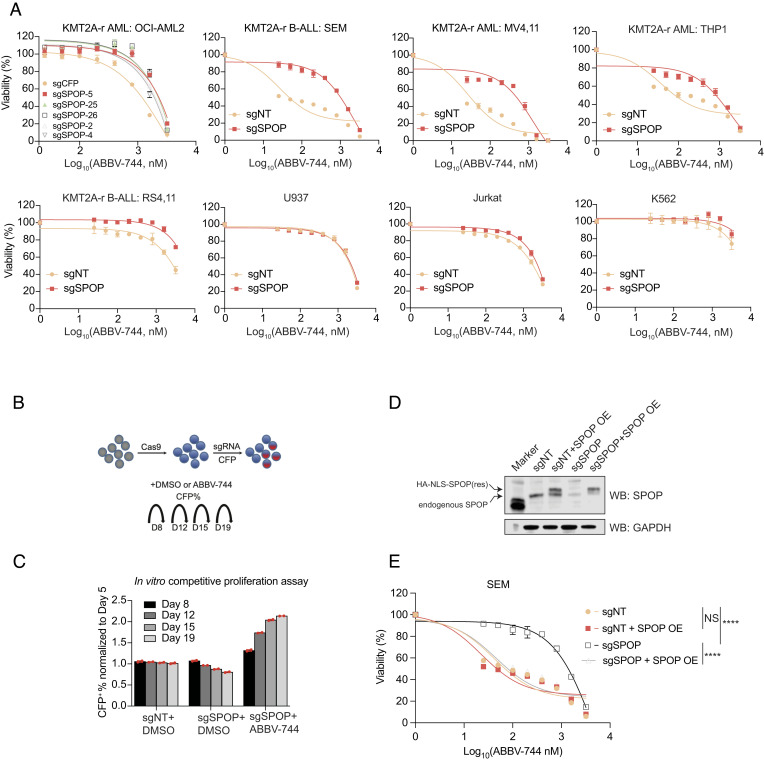
BETi-resistance phenotype is associated with SPOP deficiency in leukemia cell lines. (*A*) An MTT assay was conducted to validate the ABBV-744–resistant phenotype by using candidate sgRNAs against *SPOP* (sgSPOP-2, -4, and -5) and 2 newly designed sgRNAs (sgSPOP-25 and sgSPOP-26) in OCI-AML2 cells, other KMT2A-r (SEM, MV4,11, THP1, and RS4,11), and non-KMT2A-r (U937, Jurkat, and K562) leukemia cell lines. sgNT: negative control sgRNA. (*B*) Flow diagram of the competitive assay upon drug treatment based on CFP^+^% quantification. (*C*) The competitive assay was conducted in sgNT- and sgSPOP-treated cells maintained in culture with DMSO and in sgSPOP-treated cells in culture with ABBV-744 for 19 d. Flow cytometry was performed to quantify the drug-resistant cells. Two replicates are shown. (*D*) The lentiviral expression of SPOP cDNA was transduced into the sgNT- and sgSPOP-treated cells, followed by immunoblotting to confirm the expression. (*E*) MTT assay was conducted to examine the rescue effect upon ABBV-744 treatment. OE: overexpression. Statistical analysis was carried out by AUC followed by a *t* test. ***P < 0.001, ****P < 0.0001, n.s., not significant.

To validate the CRISPR screen results, we designed 5 sgRNAs to target different exons of the *SPOP* gene in the KMT2A-r AML cell line OCI-AML2 expressing Cas9. Efficient CRISPR targeting of each sgRNA was confirmed by indel PCR followed by ICE and TIDE-seq analysis (30). MTT assay was then conducted to quantify cell proliferation against drug treatment and CRISPR editing. After treating cells with ABBV-744 for 4 d, we observed a consistent drug-resistant phenotype in all 5 sgRNA-targeted bulk populations, compared to the nontargeting control sgRNA (sgNT) (Fig. 2*A*). We then performed an MTT assay in additional KMT2A-r and non-KMT2A-r leukemia cell lines to test the generalizability of the findings. To that end, we infected the KMT2A-r B-ALL cell lines SEM and RS4,11 (carrying a KMT2A-AF4 fusion), the KMT2A-r AML cell lines MV4,11 (carrying a KMT2A-AF4 fusion), and THP1 (carrying a KMT2A-AF9 fusion) with lentiviral Cas9 and a sgRNA against SPOP (sgRNA-5). Compared to the sgNT control, *SPOP* depletion significantly induced drug resistance against ABBV-744. In contrast, none of the non-KMT2A-r leukemia cell lines JURKAT, K562, or U937 demonstrated apparent difference between sgNT and sgSPOP groups (Fig. 2*A* and *SI Appendix*, Fig. S2).

To further characterize the drug-resistance phenotype, we conducted an in vitro competitive proliferation assay, as described previously (31) (Fig. 2*B*). OCI-AML2 Cas9-expressing cells were infected with lentiviral sgNT or sgRNA-5 (throughout the study) against *SPOP* coupled with constitutive expression of cyan fluorescent protein (CFP). By controlling the titration of virus infection, the CFP^+^ cells were about 50% at the initial stage for sgSPOP and sgNT groups. The CFP^+^ portion was then monitored every 3 to 4 d until day 19 by flow cytometric analysis. As expected, when normalized to the baseline, the CFP^+^% remained the same when control sgNT was used. However, *SPOP*-knockout cells exhibited mild survival disadvantage upon DMSO treatment for 19 d, indicating that *SPOP* is required for leukemia cell survival. By contrast, when ABBV-744 was added to the cell culture, *SPOP*-KO (CFP^+^) cells were notably enriched over time (Fig. 2*C*). Consistently, we detected significant enrichment of frameshift mutations of *SPOP* in the pooled population of OCI-AML2 at the ABBV-744 treatment endpoint (*SI Appendix*, Fig. S3). This drug-resistance phenotype was almost entirely reversed by ectopic expression of *SPOP* cDNA (a sgRNA-resistant mutant form) in the *SPOP*-knockout cells, confirming that the drug-resistant phenotype is selectively dependent on *SPOP* deficiency (Fig. 2 *D* and *E*). Collectively, these data demonstrated the *SPOP*-mediated BETi resistance in KMT2A-r leukemia cells.

### Characterization of the BETi-Resistance Phenotype Associated with SPOP In Vivo.

**Fig. 3.**
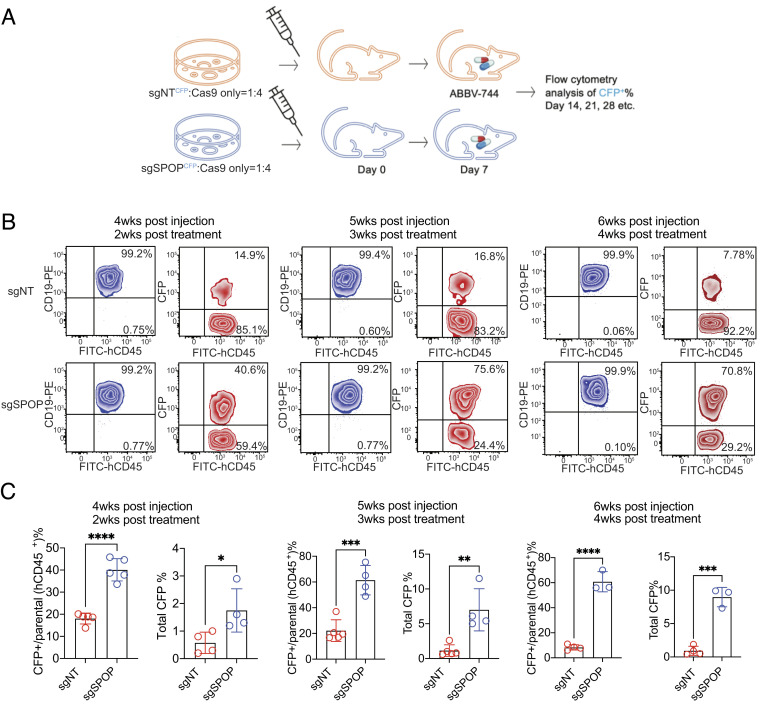
BETi resistance is associated with SPOP deficiency in vivo. (*A*) Schematic diagram for in vivo competitive growth assay. Cas9-expressing KMT2A-r SEM cell lines were transduced with sgNT-CFP control or sgSPOP-CFP gRNA and were premixed with Cas9-only cells (no CFP) at the ratio of 1:4, respectively. Mixed cells were injected into NSG mice, and ABBV-744 was administered daily by oral gavage. Flow cytometry was performed to measure the cell growth of each cell population in mouse peripheral blood. (*B*) *SPOP-*knockout cells are more resistant to ABBV-744 treatment in vivo. The *Top* panels represent the sgNT-CFP/Cas9-only SEM cell populations, and the *Bottom* panels represent the sgSPOP-CFP/Cas9-only SEM cell populations in mouse peripheral blood. The blue density plots show the human leukemia cell population, and the red density plots show CFP^+^ cells in the human leukemia cell population. (*C*) SEM cell growth in vivo. Flow cytometry analysis quantified the overall growth of sgNT-CFP or sgSPOP-CFP cells in vivo under ABBV-744 drug selection. Statistical analysis was carried out by the unpaired *t* test. *P < 0.05, **P < 0.01, ***P < 0.001, ****P < 0.0001.

Inspired by the consistent BETi-resistance phenotype of SPOP-deficient cells observed in multiple KMT2A-r leukemia cell lines in vitro, we then characterized the resistance phenotype in vivo. To that end, we established a cell-derived xenograft (CDX) model by transplanting the KMT2A-r B-ALL SEM cell line into immunocompromised NOD.Cg-*Prkdc^scid^ Il2rg^tm1Wjl^*/SZJ mice (NSG mice). The sgNT- or sgSPOP-targeted CFP^+^ SEM cells were premixed with Cas9-only cells (no CFP) at a ratio of 1:4 (~20% CFP^+^ vs. 80% CFP^-^), respectively. About 2 million cells of each group were injected into each of the five female NOD scid gamma (NSG) mice, followed by daily and oral delivery of ABBV-744 (4.7 mg/kg). Blood samples were collected by retro-orbital blood sampling at 2-, 3-, and 4-wk posttreatment, and CFP*^+^* percentages were calculated by flow cytometric analysis (Fig. 3*A*). The SEM cells expressing a CFP-linked sgSPOP were significantly enriched from 20% (start point) to 40% at 2 wk and 75% at 3 wk after completion of ABBV-744 treatment in NSG mice, as evidenced by a dramatic increase of the total CFP^+^ fraction in the engrafted CD45^+^ human cells. In contrast, the SEM cells expressing sgNT maintained the same percentage of CFP^+^ cells as the parental sgNT-targeted SEM cells (Fig. 3 *B* and *C*). Together, these results prove that the loss of SPOP can induce BETi resistance in vivo and lead to leukemia cell expansion under BETi exposure.

### Identification of Possible Substrates of SPOP That Are Responsible for BETi Resistance.

**Fig. 4.**
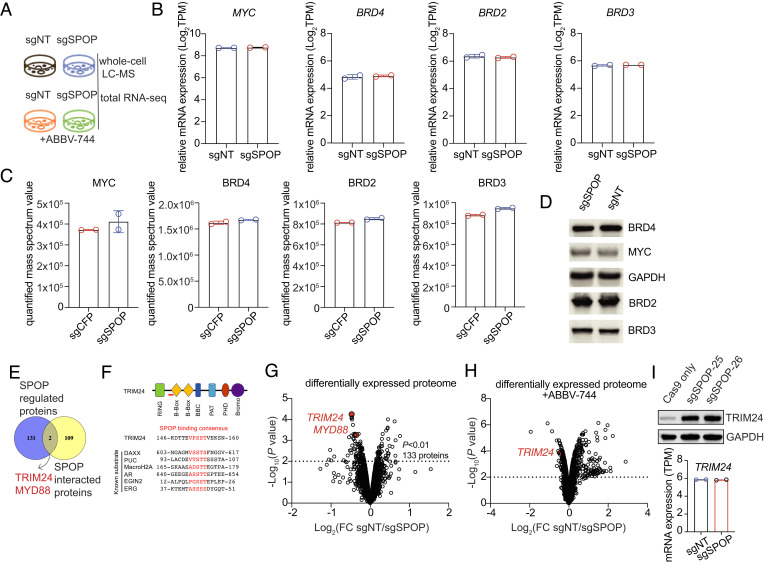
Identify the downstream targets of SPOP. (*A*) Schematic of the experimental design to identify SPOP’s substrates using whole-cell proteome and total RNA-seq. (*B*) RNA expression of SPOP’s known downstream target genes. FPKM (fragments per kilobase per million sequenced reads) values were derived from total RNA-seq with two replicates. (*C*) Quantified protein expression of SPOP’s known downstream target genes. The relative expression ratio was derived from whole-cell LC-MS with two replicates. (*D*) Immunoblotting was conducted to validate the protein levels of MYC and BRDs upon *SPOP* knockout in OCI-AML2 cells. (*E*) Venn diagram of differentially expressed proteins upon *SPOP* knockout compared with SPOP-interacted proteins. (*F*) Illustration of SPOP’s binding-consensus motif in TRIM24 peptide. (*G*) Volcano plot of differentially expressed proteins between *SPOP*-knockout and controls cells, with a *P* value cutoff of 0.01. (*H*) Volcano plot of differentially expressed proteins between *SPOP*-knockout and controls cells upon ABBV-744 treatment, with a *P* value cutoff of 0.01. (*I*) *TRIM24* mRNA and protein levels were quantified against *SPOP* knockout in OCI-AML2 cells.

Multiple lines of evidence support the hypothesis that SPOP is vital as a tumor suppressor or oncogene, depending on the context (32–35). SPOP usually acts as a substrate-adaptor protein of a cullin-3-RING ubiquitin ligase and recruits substrates to ubiquitination modification (36). Our whole-genome and posttranslational regulator CRISPR screens identified SPOP and CUL3 (*SI Appendix*, Table S1) as the top hits responsible for ABBV-744 resistance in OCI-AML2 cells. Based on these observations, we hypothesized that the loss of SPOP stabilizes specific functional substrate proteins, which confers drug resistance. In prostate cancer, BRD4 and MYC are direct substrates of SPOP, and the stabilization of BRD4 is directly linked to BETi resistance in prostate cancer cells carrying *SPOP* mutations. To test whether SPOP targets BRD4 and MYC directly to regulate MYC pathways and BETi response in KMT2A-r leukemia cells (34), we conducted total RNA-seq and whole-cell mass spectrometry assays to quantify the RNA and global protein abundance in sgNT- and sgSPOP-targeted cells, with or without ABBV-744 treatment (Fig. 4*A* and *SI Appendix*, Table S3). Surprisingly, when *SPOP* was knocked out, the mRNA and the protein levels of MYC, BRD2, BRD3, and BRD4 remained unchanged (Fig. 4 *B* and *C*), which is different from the typical SPOP/MYC regulation axis observed in a non-KMT2A-r ALL Nalm6 cells (*SI Appendix*, Fig. S4). These data are consistent with immunoblotting results using specific antibodies against each protein (Fig. 4*D*).

To determine which novel substrate proteins of SPOP might be associated with BETi resistance, we compared proteins up-regulated in the SPOP-KO setting by whole-cell mass spectrometry assay (133 at the cutoff *P* value = 0.01) with SPOP-interacting proteins identified from the protein database BioGRID (111 in human). Two overlapping proteins, TRIM24 (tripartite motif–containing protein) and MYD88 (myeloid differentiation primary response 88) were considered possible hits (Fig. 4*E*), both of which were previously identified as SPOP substrates (37–39). However, whether TRIM24 or MYD88 plays a role in BETi resistance in leukemia has not been well determined.

When we combined the differentially expressed proteins between *SPOP*-knockout and wild-type (WT) *SPOP* settings, with or without ABBV-744 treatment, we narrowed the candidates to the shared substrate TRIM24. TRIM24 expression is up-regulated by sgSPOP, with or without ABBV-744 treatment, and the level of MYD88 increased only in sgSPOP-targeted cells without ABBV-744 treatment (Fig. 4 *G* and *H*). By amino acid sequence alignment, we identified an SPOP-binding consensus sequence in TRIM24 (Fig. 4*F*). We further confirmed the dramatic upregulation of TRIM24 in 2 sgSPOP-targeted cell lines by immunoblotting (Fig. 4*I*); however, the mRNA expression level remained unchanged. Together, these data support that loss of SPOP stabilizes TRIM24 in BETi-resistant cells and confirm TRIM24 is a possible substrate that mediates BETi resistance in response to *SPOP* deficiency. To test this hypothesis, two sgRNAs targeting *TRIM24* (coupled with zeocin resistance) were delivered into sgSPOP-targeted SEM cells (associated with puromycin resistance), followed by dual-antibiotic selection. SPOP and TRIM24 were efficiently knocked down by the corresponding sgRNAs in a bulk population (*SI Appendix*, Fig. S5*A*). As a result, the partial restoration of drug sensitivity against ABBV-744 was seen in the population cotargeted with sgSPOP and sgTRIM24-1 at all dosages of ABBV-744 treatment (*SI Appendix*, Fig. S5*B*). It is possible that cells cotargeted by TRIM24 and SPOP gRNAs exhibit a survival disadvantage upon BETi treatment compared to cells targeted for SPOP alone. Incomplete CRISPR knockout of TRIM24 in the bulk sgSPOP population may result in the selective loss of TRIM24 targeted cells upon BETi treatment over time, which could explain the partial rescue observed. Alternatively, it may also suggest other downstream target genes other than TRIM24 may play a role in this process. Comprehensive analysis to figure out the detailed mechanism is needed in future studies.

### CRISPR Screen to Identify Potentially Targetable Pathways in BETi-Resistant Cells.

**Fig. 5.**
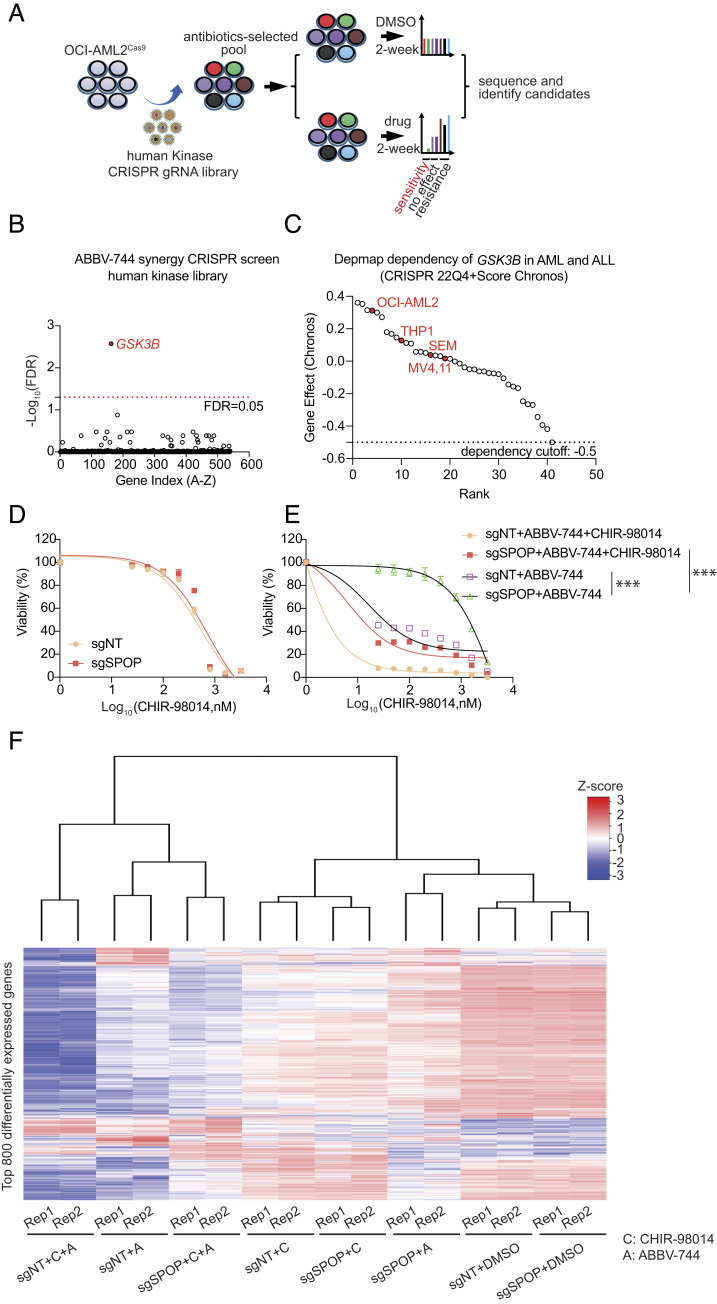
CRISPR screen reveals that GSK3 inhibition resensitizes the response of *SPOP*-knockout cells to BETi. (*A*) Schematic of a loss-of-function human kinase CRISPR screen to identify a targetable pathway to cope with BETi resistance using KMT2A-r AML OCI-AML2 cells. (*B*) The enrichment score of several sgRNAs against each gene was combined by the MAGeCK algorithm. The top synergy-associated candidate gene was highlighted based on the Log_10_(FDR) with a cutoff of 0.05. (*C*) The dependency score of GSK3β among human AML and ALL cell lines were collected from the Depmap database. An MTT assay was conducted using *SPOP*-knockout and control (sgNT) cells treated with the GSK3i CHIR-98014 alone in (*D*) and in combination with ABBV-744 in (*E*) using SEM cells. Statistical analysis was carried out by AUC followed by a *t* test. ***P < 0.001. (*F*) An unsupervised heat map of total RNA-seq analysis characterizes the molecular response against single-and dual-inhibitor treatment focusing on the top 800 differentially expressed genes. Two replicates of each SEM cell line sample upon treatment were characterized.

To identify targetable pathways that overcome SPOP-associated BETi resistance, we took advantage of the powerful CRISPR-mediated loss-of-function screen that we previously established (Fig. 5*A*). We utilized the OCI-AML2 cell line stably expressing Cas9 and a commercial CRISPR/Cas9 sgRNA library targeting about 496 human kinase genes to identify functional kinase pathways in BETi-resistant cells. In brief, the CRISPR/Cas9 library–targeted pools were cultured with DMSO and ABBV-744 (100 nM) for 2 wk and assessed by next-generation sequencing and sgRNA-deconvolution, per the MAGeCK algorithm. We identified *GSK3*β as a synergistic gene associated with sensitivity to ABBV-744 treatment (Fig. 5*B* and *SI Appendix*, Table S4). Interestingly, *GSK3*β is not a survival dependency gene in human leukemia cell lines, including OCI-AML2 (Fig. 5*C*). Meanwhile, we also conducted gene set enrichment analysis (GSEA) of the publicly available transcriptome from TRIM24-targeted cells and identified the GSK3α pathway significantly affected by TRIM24 inhibition (*SI Appendix*, Fig. S5 *C* and *D*).

Data collected from the CRISPR screens and GSEA support that the GSK3α/β pathway represents a promising target to overcome SPOP-associated BETi resistance. To confirm, we selected a potent GSK3 inhibitor (GSK3i), CHIR98014, to test the combined effect of CHIR98014 with ABBV-744 treatment in SPOP-deficient cells. As expected, our data showed no differences in cell viability between SPOP-deficient cells and controlled SEM cells with CHIR98014 treatment alone (Fig. 5*D*). However, the cell viability in an *SPOP*-knockout setting was significantly reduced when ABBV-744 and CHIR98014 were simultaneously administrated, suggesting a potential synergistic effect (Fig. 5*E*). The transcriptome characterization of samples treated with single drug or combination consistently confirmed the synergistic effect of ABBV-744 and GSK3i led to the most significant differential gene expression (Fig. 5*F* and *SI Appendix*, Table S3).

### Combination Therapy Using GSK3i and BETi to Treat PDXs In Vivo.

The hypothesis-driven and unbiased analyses identified GSK pathways as a plausible target to overcome resistance. Therefore, we addressed whether targeting the GSK3 pathway restores BETi sensitivity in SPOP-deficient cells in vitro, and then, we tested the cotargeting effect by using the in vivo transplantation model. In brief, sgNT- and sgSPOP-targeted bulk SEM cells (n = 2 million cells/mouse) were injected into host NSG mice via the tail vein. The mice were then split into six groups based on drug combinations (*SI Appendix*, Fig. S6*A*). After the leukemia cell injection, a daily oral dose of ABBV-744 (4.7 mg/kg body weight) was given. Blood samples were collected by retro-orbital blood withdrawal at 3- and 5-wk posttreatment, and the percentages of human CD45+ cells were calculated by flow cytometric analysis (*SI Appendix*, Fig. S6 *B* and *C*). Consistent with the in vitro results, the synergistic effect of GSK3i and ABBV-744 was observed in SPOP-deficient cells in NSG mice, as evidenced by a dramatic decrease in the total human CD45+ cell fraction in peripheral blood.

**Fig. 6.**
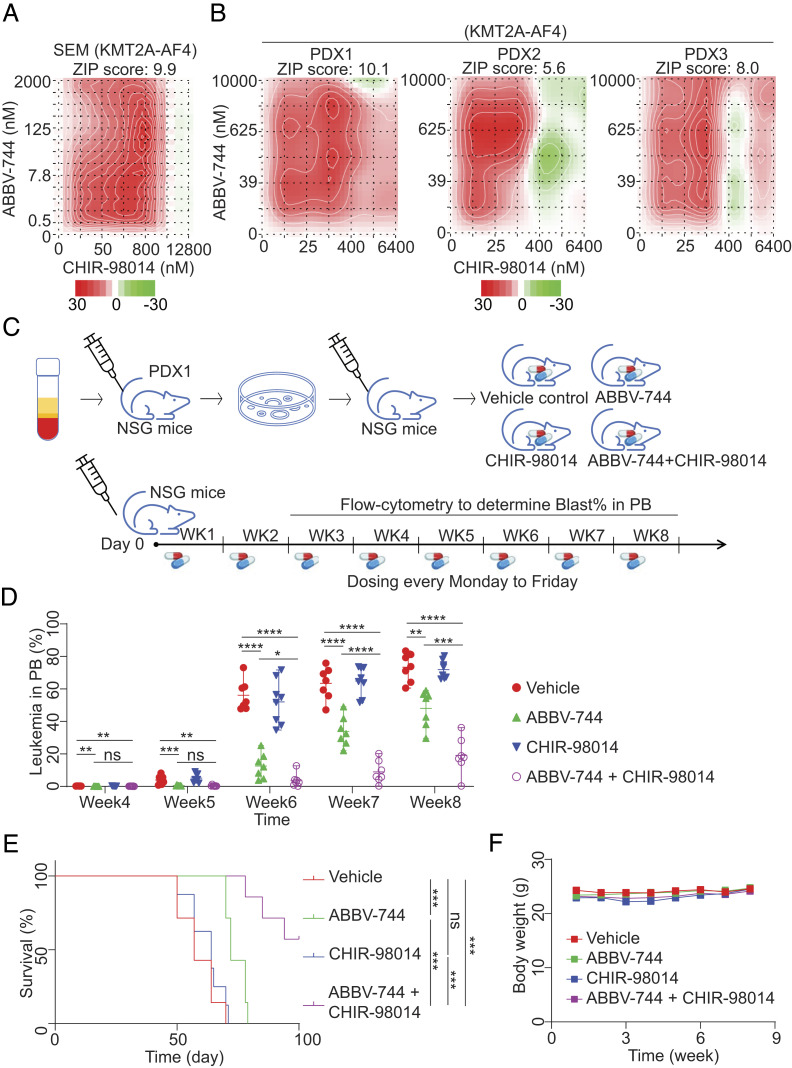
In vitro and in vivo combinatory effects of GSK3i and BETi in patient-derived xenografts. (*A* and *B*) In vitro combination of BETi (ABBV-744) and GSK3i (CHIR-98014) was assessed by cell viability CTG assay for SEM cell line (*A*) and high-content imaging assay for PDX cells (*B*, details are described in the *Methods*). Synergy index ZIP score was estimated using Synergyfinder. The color bar indicates synergy activity (red: high; green: low). (*C*) Schematic diagram for in vivo combination therapy assay. Primary KMT2A-r ALL cells were injected into NSG mice for expansion. Upon engraftment, PDX cells were isolated from NSG mice and injected into other NSG mouse recipients, followed by dosing of BETi, GSK3i, or the combination. A weekly flow cytometry assay determined leukemia burden in mouse peripheral blood (PB). (*D*) Leukemia burden in mouse peripheral blood. Human leukemia burden was measured by flow cytometry. Each treatment arm included 7 to 8 mice. The *P* value was determined by a 2-tailed *t* test. (*E*) Survival analysis of mice treated with BETi, GSK3i, or combination therapy. The mantel-Cox test was used to determine the *P* value. (*F*) Body weight of mice treated with BETi, GSK3i, or combination therapy was measured. The health status of each mouse treated with vehicle, BETi, GSK3i, or combination therapy was monitored daily, and body weight was measured weekly. No mice treated with BETi, GSK3i, or combination therapy showed severe symptoms or dramatic weight loss. *P < 0.05, **P < 0.01, ***P < 0.001, ****P < 0.0001; n.s., not significant.

Given that human cell lines often develop additional acquired mutations during their long-term culture in vitro that may not wholly reflect drug treatment response, we repeated this experiment in the PDX models. We first characterized three newly established human KMT2A-r B-ALL PDX lines (carrying KMT2A-AF4 fusion) by the transplantation model in NSG mice (*SI Appendix*, Fig. S7*A*). By conducting an ex vivo drug-sensitivity assay, we confirmed the favorable response of each PDX sample against single-agent treatment (*SI Appendix*, Fig. S7 *A*–*C*). Moreover, we observed a robust synergistic effect with ABBV-744 and CHIR98014 cotreatment, like in SEM cells (Fig. 6 *A* and *B*). Inspired by these data, we transplanted the PDX cells into NSG mice, followed by the drug treatment, including vehicle, ABBV-744, CHIR98014, and a combination thereof, 1 day after the leukemia cells were injected (Fig. 6*C*). We calculated the percentage of human CD45+ cells in the peripheral blood to determine leukemia cell growth after 4 wk posttransplantation. Starting from 5 wk, we observed comparable leukemia cell expansion between vehicle and CHIR98014-alone groups. We consistently detected moderate proliferation inhibition in the ABBV-744 treatment group and significant growth retardation in the combination-treated group at each time point (Fig. 6*D*). Meanwhile, survival was modeled using the Kaplan–Meier method. A considerable extension was observed in the combination treatment group (Fig. 6*E*). More importantly, we did not observe any weight loss upon drug treatment, suggesting the likelihood of safety of the current treatment regimen is likely safe (Fig. 6*F*), which holds great promise for future therapy in the clinic setting. Finally, upon dual drug treatment, we purified PDX1 leukemia cells by flow cytometry against CFP and CD45+ from the relapsed mice. We performed the drug sensitivity test in vitro compared with the parental PDX1 cells. Notably, the dual drug-resistant PDX1 cells from in vivo no longer responded to any single drug (*SI Appendix*, Fig. S7*D*).

## Discussion

Targeting BD proteins by BETi is a promising therapeutic approach for numerous types of cancer. However, the molecular mechanisms of BETi response and resistance pathways in KMT2A-r leukemias and other cancers remain unclear. In this study, we found a role of SPOP in acquired BETi resistance of KMT2A-r leukemia that could not be identified through genomic sequencing of large patient cohorts without BETi drug treatment. We studied the SPOP-mediated BETi resistance phenotype and provided an alternative therapeutic strategy to overcome BETi resistance in SPOP-deficient cells by targeting the GSK3 pathway. We also identified the synergistic effect of BETi and GSK3i in KMT2A-r leukemia by utilizing ex vivo and in vivo mouse/human models for therapeutic innovation.

The second-generation BETi ABBV-744 selectively targets the BD2 of BET proteins (14, 21). Compared with first-generation BETis, which target both BD1 and BD2, ABBV-744 exhibits greater potency and less toxicity in prostate cancers (14, 21). ABBV-744 shows therapeutic efficacy in vitro and in vivo against a small set of AML cell lines and patient-derived primary AML cells as monotherapy, in some cases causing sustained regression (40). The molecular mechanisms mediating drug response and resistance of these BETis remain poorly understood. Because ABBV-744 is still being tested in clinical trials and little is known about drug resistance, there is an urgent need to conduct such studies. To this end, we developed a powerful combinatorial approach using genome editing and disease-relevant in vivo models and identified SPOP as the top candidate.

Previous studies have illustrated that SPOP functions as a substrate adaptor of cullin 3–based E3 ligase with crucial roles in prostate cancer, endometrial cancer, and gastric cancer development (41–46). SPOP consists of an N-terminal MATH domain, an internal Broad-Complex, Tramtrack, and Bric à brac/ poxvirus and zinc finger (BTB/POZ) domain, an internal BACK domain (with a smaller three-box domain), and a C-terminal nuclear-localization sequence. SPOP-Cul3 recognition occurs primarily through the BTB domain, which regulates proper cellular protein degradation in many solid tumors. Somatic *SPOP* mutations have been frequently identified in patient samples, with hotspot mutations occurring at the N-terminal MATH domain, including Y87, F102, F133, and W231 (41, 42). The MATH domain is responsible for the direct interaction with substrate proteins through the substrate-binding consensus motifs. SPOP plays an essential role in various cellular processes via specific targeting of proteins for ubiquitination and subsequent proteasomal degradation during cancer development. Dysregulation of SPOP-mediated proteolysis can directly target BRD4 and MYC (38, 47). Therefore, elevated expression of BRD4 and MYC confer JQ1 resistance in prostate cancers. Although genomic *SPOP* mutation is rarely observed in human leukemia, our preliminary data identified *SPOP* as an essential gene in BETi-resistant leukemia cells; however, BRD4 and MYC were not direct substrates. Instead, our data suggested that BETi resistance in *SPOP*-knockout cells occurred by other possible substrates and the downstream signaling pathway. In addition, whether SPOP can play a role in BETi resistance in noncanonical function independent of protein degradation is not clear. Therefore, the detailed molecular mechanism associated with our reported phenotype requires further investigation.

Many combination therapy regimens have recently been developed to target high-risk leukemia, including the BCL2i/BETi (40), CDK7i/BETi (25), CBP/P300/BETi (48), and JMJD6i/BETi (49). Unlike these combination treatments, the effects of which are based on the efficacy seen in monotherapy, CHIR-98014 alone showed no effect on the survival of KMT2A-r leukemia cells in vitro or in vivo. However, we observed a significantly improved therapeutic effect when we added CHIR-98014 to ABBV-744 therapy. Additionally, we observed no severe toxicity in the combination-treated PDX mice, suggesting tolerable toxicity. A more systematic toxicology evaluation is needed in the future.

In summary, we discovered an unappreciated role of *SPOP* in acquired BETi resistance of KMT2A-r leukemia that cannot be identified through genomic sequencing of large patient cohorts without BETi drug treatment. The in vivo mouse/human models and state-of-the-art techniques, including CRISPR gene editing, functional CRISPR screen, pharmacological perturbation, and genome-wide approaches for systematical characterization of the regulation network, identified a promising combination treatment regimen of using ABBV-744 and CHIR-98014. Although the methods used were designed to study acquired BETi resistance in KMT2A-r leukemia, they can be broadly applied to study acquired resistance in other disease types.

## Methods

### Cell Culture.

SEM (ACC-546, DSMZ), OCI-AML2 (ACC-99, DSMZ), MV4,11 (ACC-554, DSMZ), THP1 (ITB-202), U937 (CRL-1593.2), K562 (CCL-243), and Jurkat (TIB-152) were maintained in RPMI-1640 medium (Lonza) containing 10% fetal bovine serum (FBS) (HyClone) and 1% penicillin/streptomycin (Thermo Fisher Scientific); hTERT mesenchymal stem cells (MSCs; ABM, #T0529) were maintained in MSC culture medium made from RPMI-1640 (Gibco, #11875093), 10% heat-inactivated FBS (Gibco, #10082-147), and 1 μM hydrocortisone (Sigma, #H0396); all cells were incubated at 37 °C, 5% CO_2_ atmosphere, and 95% humidity. The basal medium for maintaining 293T cells in culture was DMEM (HyClone). All passages of cells used in this study were *mycoplasma*-free. Cell identity was confirmed by Short Tandem Repeats (STR) analysis.

### Vector Construction.

The Lenti-Cas9-Blast plasmid (#83480) and the Lenti-Guide-Puro plasmid (#52963) were purchased from Addgene. The Lenti-H3-sgRNA-library (#133914) and Lenti-Kinase-sgRNA-library (#117725) were initially generated by the laboratories of Shirly Liu and Christopher Vakoc. We purchased both libraries from Addgene. The Lenti-posttranscriptional library was developed by our lab and deposited at Addgene (#171531). For candidate validation of CRISPR screen and competitive proliferation assay, sgRNA sequences against SPOP were cloned into an in-house–made Lenti-Guide-Puro-IRES-CFP vector through BsmBI sites. The sgRNA-resistant SPOP cDNA was cloned into the pCDH-MND-MCS-EF1-RFP vector.

### PDXs and Ex Vivo Drug-Sensitivity Assay.

ALL PDXs were developed using deidentified primary samples obtained at St. Jude Children’s Research Hospital or MD Anderson Cancer Center. The study was approved by the Institutional Review Board of St. Jude Children’s Research Hospital (IRB protocol No. XPD 14-992). NOD.Cg-*Prkdc^scid^ Il2rg^tm1Wjl^*/SzJ NSG mice were used for PDX development. All animal studies were approved by the Institutional Animal Care and Use Committee of St. Jude Children’s Research Hospital. For each case, primary human leukemia cells were injected into 8- to 12-week-old female NSG mice through the tail vein (2 million cells/mouse, resuspended in 200 μL sterile PBS). The health status of all injected mice was monitored every day. Starting at 2 wk after injection, peripheral blood was obtained via retro-orbital sampling and subjected to flow cytometry to determine the level of human leukemia (CD45+, CD19+) every 2 wk. Mice were killed when leukemia reached 80% in peripheral blood, or the animal became moribund. After the mice were killed, human leukemia cells were harvested from the spleen and bone marrow and enriched by negative selection using an immunomagnetic isolation kit (Stemcell, #19849). The drug response of primary human leukemia cells was evaluated using a coculture system and high-content imaging. The hTERT-immortalized MSCs were first seeded in a 384-well plate (PerkinElmer, #6057308) at a density of 2,500 cells per well in 25 μL MSC medium 24 h before PDX sample preparation. After 24 h, the MSC medium was removed, and wells were washed with an AIM V medium (Gibco, #12055-083). Sorted leukemia cells were added at 25,000 cells per well to the stromal cell layer in 40 μL AIM-V medium, and 10 μL drug solution was prepared in the same medium. Triplicate samples were included for each drug concentration/combination. After 96-h incubation at 37 °C with 5% CO_2_, the cells were harvested and stained with CyQUANT direct cell proliferation assay stain (Invitrogen, #C35011). After 15 to 30 min of incubation at 37 °C with 5% CO_2_, the assay plate was placed in the high-content imaging analysis system (PerkinElmer Operetta CLS, #HH16000000). Cell viability data were analyzed by Harmony high-content imaging and analysis software (version 4.9).

### In Vitro Drug Sensitivity Assay in Cell Lines.

An MTT assay was used to determine the drug sensitivity of human leukemia cell lines. On day 0, cells were collected and resuspended in RPMI1640 (Lonza, #12-918F), supplemented by 10% FBS. A 100-μL cell suspension was then plated on round-bottom 96-well plates (10,000 to 24,000 cells/well, depending on the cell line). Drug stock was thawed at room temperature, and a working solution was made by serial dilution with medium and added to the cell suspension in 96-well plates. Vehicle control and blank (medium control) were set appropriately. After a 4-d incubation at 37 °C, 5% CO_2_, 10-μL MTT solution (Sigma, M5655-1G) was added to each well. Cells were then incubated at 37 °C, 5% CO_2_ for 4 to 6 h. About 100 μL isopropanol (Sigma, Cat# 59304) was added, and the mixture was kept at room temperature for 5 min before measuring the absorbance at 562 nm.

### In Vivo Competition Assay and Drug Efficacy Evaluations.

For the in vivo competition assay, SEM cells, with or without *SPOP* knocked out, were premixed and injected into NSG mice, followed by daily injection of ABBV-744 starting 1 d posttumor inoculation. ABBV-744 was given at 4.7 mg/kg via oral gavage. Retro-orbital blood was collected weekly and subjected to flow cytometric analysis to determine the ratio of *SPOP*^WT^ to *SPOP*^KO^ cell populations. Leukemia burden was also monitored weekly by flow cytometry, and mice with a human blast percentage over 80% in blood or signs of being moribund were considered life terminal in survival analysis and euthanized.

For the in vivo efficacy test of ABBV-744 in SEM cells, we injected *SPOP*^WT^ and *SPOP*^KO^ cells into NSG mice through tail vein injection and started daily ABBV-744 treatment at 4.7 mg/kg. Beginning 2 wk after injection, peripheral blood was obtained weekly by retro-orbital sampling, and flow cytometric analysis was performed to determine the level of human leukemia (CD45+, CD19+). Mice with human leukemia percentage over 80% in blood or signs of being moribund were considered life-terminal in survival analysis and euthanized.

For in vivo efficacy evaluation of the combination of ABBV-744 and CHIR-98014, KMT2A-r B-ALL PDX cells were injected into female NSG mice between 8 and 12 wk of age through the tail vein (2 million cells/mouse, resuspended in 100 μL sterile PBS). The health status of all injected mice was monitored daily. ABBV-744, CHIR98014, vehicle control, or the combination of ABBV-744 and CHIR-98014 was given to leukemia-bearing NSG mice starting at 1 d postinjection. Each drug or combination was administered once daily through oral gavage (ABBV-744 was given at 4.7 mg/kg, and CHIR-98014 was given at 15 mg/kg). Starting 2 wk after injection, peripheral blood was obtained by retro-orbital sampling weekly and subjected to flow cytometry to determine the level of human leukemia (CD45+, CD19+). Mice with a human blast percentage over 80% in blood or signs of being moribund were considered life terminal in survival analysis and euthanized.

### PDX and Consent Information.

ALL PDXs were developed using primary samples obtained at St. Jude Children’s Research Hospital or MD Anderson Cancer Center. Patients were provided informed consent for the research use of their specimens, which was approved by the Institutional Review Board of St. Jude Children’s Research Hospital and MD Anderson. All samples were deidentified prior to PDX generation. NOD.Cg-Prkdcscid Il2rgtm1Wjl/SzJ NSG mice were used for PDX development. All animal studies were approved by the Institutional Animal Care and Use Committee of St. Jude Children’s Research Hospital (protocol number 624).

### CRISPR Screen and Analysis.

The OCI-AML2 cell line was overexpressed with lentiviral Cas9, followed by infection with pooled sgRNA library at low M.O.I. (~0.3). Infected cells were selected by blasticidin and puromycin and later with drug treatment for about 14 d (ABBV-744: 100 nM, JQ1: 100 nM, dBET1: 10 nM). The sgRNA sequences were recovered by genomic PCR analysis and deep sequencing using MiSeq for single-end 150-bp read length (Illumina). The primer sequences used for cloning and sgRNA sequences are described in *SI Appendix*, Table S2. The high-titer lentivirus stocks were generated in 293T cells as previously described (50). The raw FASTQ data were debarcoded and mapped to the original reference sgRNA library. The differentially enriched sgRNAs were defined by comparing normalized counts between DMSO and drug-treated bulk populations. Two independent replicate screenings were performed for each drug treatment group. Normalized counts for each sgRNA were extracted and used to identify differentially enriched sgRNA by DESeq2 (51). The combined analysis of 7 sgRNAs against each human transcription factor was conducted using the MAGeCK algorithm (52). Candidate rank was based on the Log_10_(FDR) for resistance phenotype. Detailed screening results are included in *SI Appendix*, Table S1.

### Flow Cytometry.

Suspension-cultured leukemia cells were collected by centrifugation at 800 × g, filtered through a 70-µm filter, and sorted for CFP^+^ on a BD FACS Aria III flow cytometer with negative control. DAPI staining was conducted before sorting to exclude dead cells.

### Immunoblotting.

 Cell lysates were prepared using Radioimmunoprecipitation assay (RIPA) buffer, followed by SDS-PAGE (Thermo Fisher Scientific), and transfer of the lysate to a PVDF membrane per the manufacturer’s (Bio-Rad) protocols at constant 100 V for 1 h. After blocking incubation with 5% nonfat milk in TBS-T (10 mM Tris, pH 8.0, 150 mM NaCl, 0.5% Tween-20) for 1 h at room temperature, the membrane was incubated with antibodies against GAPDH, SPOP, TRIM24, MYC, GSK3α, GSK3β, BRD2, BRD3, and BRD4 at 4 °C for 12 h with gentle shaking (*SI Appendix*, Table S2). Membranes were washed 3 times for 30 min and incubated with horseradish peroxidase-conjugated anti-mouse or anti-rabbit antibodies for 2 h at room temperature. Blots were washed with TBS-T 3 times for 30 min and developed with the ECL system (Amersham Biosciences) per the manufacturer’s instruction.

### RNA-seq and Data Analysis.

Total RNA was extracted by TRIzol (Thermo Fisher Scientific, 15596026) from replicate samples of cells treated with DMSO, CHIR98014, ABBV-744, or a combination thereof. About 200 ng total RNA was treated using Kapa rRNA depletion reagents to remove ribosomal RNA and then converted into cDNA libraries using Kapa RNA HyperPrep Kit with RiboErase (HMR). After end repair, dA-tailing, and adapter ligation, each cDNA library was purified and enriched by 11 cycles of PCR amplification. All RNA-seq libraries underwent 101-cycle paired-end sequencing on the Illumina Novaseq system. Quality control filtering and adapter trimming of fastq files was performed with trim_galore. Paired-end reads were mapped by STAR(v2.7.1a) using parameters “-c -p 4 --outFilterType BySJout --outFilterMultimapNmax 20 --alignSJoverhangMin 8 --alignSJstitchMismatchNmax 5 -1 5 5 --alignSJDBoverhangMin 10 --outFilterMismatchNmax 999 --outFilterMismatchNoverReadLmax 0.04 --alignIntronMin 20 --alignIntronMax 100000 --alignMatesGapMax 100000 --outSAMmapqUnique 60 --outSAMmultNmax 1 --outSAMstrandField intronMotif --outSAMattributes NH HI AS nM NM MD --outSAMunmapped Within --outSAMtype BAM SortedByCoordinate --outReadsUnmapped None --chimJunctionOverhangMin 12 --chimSegmentReadGapMax 3 --chimMultimapNmax 10 --chimMultimapScoreRange 10 --chimNonchimScoreDropMin 10 --chimOutJunctionFormat 1 --quantMode TranscriptomeSAM GeneCounts --twopassMode Basic --peOverlapNbasesMin 12 --peOverlapMMp 0.1 --outWigType wiggle --outWigStrand Stranded --outWigNorm RPM”. Read duplication was marked with MarkDuplicates from GATK (v4.1.2.0). RSEM was used to quantify read counts per gene. Differentially expressed genes were identified using the generated count data described above and DESeq2 (version 1.14.0). The log_2_ (fold change) values of all genes from the whole-transcriptome comparison were uploaded for GSEA. FPKM values of all genes are provided in *SI Appendix*, Table S3.

### Proteomics Analyses.

Two million OCI-AML2 cells targeted with sgNT or sgSPOP, with or without ABBV-744 treatment, each with triplicate replications, were applied for deep proteomic analyses using a well-established protocol (53–55). In brief, cells were lysed in fresh lysis buffer (50 mM HEPES, pH 8.5, 8M urea, 1× PhosStop phosphatase inhibitor, 0.5% sodium deoxycholate). Proteins were quantified by the BCA protein assay (Thermo Fisher Scientific). About 100 µg protein from each sample was digested with Lys-C (Wako, 1:100 w/w) for 2 h, followed by trypsin digestion (Promega, 1:50 w/w) overnight at room temperature after 4× dilution with 50 mM HEPES buffer. The resulting peptides from each sample were desalted, labeled with TMTpro reagents, and equally pooled. Peptides were separated into 80 fractions via a 2-h gradient and further concatenated into 40 fractions. Each fraction was dried and reconstituted in 5% formic acid for mass spectrometric analysis. Peptides were analyzed on an Orbitrap Exploris 480 mass spectrometer (Thermo Fisher Scientific) after being separated on a 20 cm × 75 µm id column packed with 1.9-µm C18 resin (Maisch GmbH, Germany) and heated at 55 °C. Peptide separation was achieved through a gradient of ~15 to 40% buffer B (0.2% formic acid, 65% CAN, 3% DMSO). The mass spectrometer was set in DDA mode with 60,000 resolution, 1 × 10^6^ AGC target, and 50-ms maximal ion time for MS1; Top 10, 1 × 10^5^ AGC target, 105-ms maximal ion time, 1-m/z isolation window, and 0.2-m/z offset, 38 NCE, and 15-s dynamic exclusion for MS_2_. Proteomic data were processed by the hybrid JUMP software suites for improving sensitivity and specificity (56, 57). Briefly, raw files were searched against the UniProt human database, and the same search and filtering parameters were applied to achieve 1% protein FDR using the target-decoy approach (58).

### Quantitative Real-Time PCR.

Total RNA was collected using TRIzol (Thermo Fisher Scientific #15596026) or Direct-zol RNA Miniprep Kit (Zymo #R2052). Reverse transcription was performed using a High-Capacity cDNA Reverse Transcriptase Kit (Applied Biosystems #4374966). Real-time PCR was performed using the FAST SYBR Green Master Mix (Applied Biosystems #4385612), per the manufacturer’s instructions. Relative gene expression was determined using the ΔΔ-CT method (59). All qPCR primers used in this study are listed in *SI Appendix*, Table S2.

### Statistical Analyses.

Statistical analyses were performed using GraphPad Prism 6.0. *P* values in Q-PCR experiments were calculated by performing a 2-tailed *t* test from 2 or 3 independent biological replicates. *P* values in the 3-(4,5-dimethylthiazol-2-yl)-2,5-diphenyl tetrazolium bromide (MTT) assay were calculated by the area under the curve (AUC) followed by a *t* test.

## Supplementary Material

Appendix 01 (PDF)

Appendix 02 (PDF)

Appendix 03 (PDF)

Appendix 04 (PDF)

Appendix 05 (PDF)

Appendix 06 (PDF)

Appendix 07 (PDF)

Appendix 08 (PDF)

Dataset S01 (XLSX)

Dataset S02 (XLSX)

Dataset S03 (XLSX)

Dataset S04 (XLSX)

## Data Availability

Raw proteomic data supporting this study’s findings are deposited at ProteomeXchange (Username: reviewer_pxd026482@ebi.ac.uk Password: oRIB3kLK). RNA-seq data were deposited at National Center for Biotechnology Information Gene Expression Omnibus (GSE201137) (60) and FPKM results were included in *SI Appendix*, Table S3. Raw data collected from CRISPR screenings were included in *SI Appendix*, Tables S1 and S4. The publicly available data sets used in this study were cited accordingly.
