# Supplementary material for: Interrogating bromodomain inhibitor resistance in KMT2A-rearranged leukemia through combinatorial CRISPR screens
Source: Proc Natl Acad Sci U S A. 2023 Apr 10;120(16):e2220134120. doi: 10.1073/pnas.2220134120 (PMC10120025; doi:10.1073/pnas.2220134120)
Supplement: Supplementary file 1 — Appendix 01 (PDF) [file pnas.2220134120.sapp1.pdf]

Supplementary Figure Legends

Figure S1. Quality control of CRISPR screen. A. Diagram of the currently available BETis and their target sites. This image was adapted from the PMID32193360. **B.** Pearson's correlation analysis was performed. The correlation score was based on total sgRNA counts and distribution upon DMSO and drug treatment (ABBV-744, JQ1, or dBET1) combined with whole-genome or post-translational CRISPR screens.

Fig. S2. Evaluation of the knockout effect of *SPOP* in human leukemia cell lines. A. Bulk population of each leukemia cell line was transduced with Cas9 and sgNT or sgSPOP and then selected by puromycin/blasticidin for three days. Cell pellets were used to perform immunoblotting using the antibody against SPOP. GAPDH was used as a loading control. **B.** A MTT assay was conducted to monitor the response upon ABBV-744 treatment when the bulk population was targeted by sgSPOP in MOLM13 cells. Immunoblotting was performed to characterize *SPOP* knockout and protein level change.

Fig. S3. Characterization of indel frequency induced by sgSPOP in OCI-AML2 cells. A. A bulk population transduced with Cas9 and sgSPOP was cultured with DMSO or ABBV-744 at 100nM for 19 days, followed by genomic PCR and TIDE-seq analysis of indel frequency. **B.** Indel frequency and detailed profiling were shown by one replicate sample targeted with sgSPOP following 19 days of culture with ABBV-744.

Fig. S4. Evaluation of the role of *SPOP* in Nalm6 cells upon BETi treatment. A. An MTT assay was conducted to monitor the response of non-targeting control and SPOP-

deficient cells to ABBV-744 treatment. **B.** Immunoblotting was performed to characterize *SPOP* knockout and protein level changes of MYC and BRD4.

Fig. S5. Preliminary study to investigate the function of TRIM24 in SPOP-mediated BETi resistance. A. Immunoblotting was conducted to examine the knockout (KO) efficiency of TRIM24 (two sgRNAs 1 and 2, Zeocin selection) and SPOP (puromycin selection). **B**. An MTT assay was conducted to monitor the response to ABBV-744 treatment in nontargeting control cells, *TRIM24*-KO bulk populations, *SPOP*-KO bulk populations, and double-knockout (DKO) SEM cells. The statistical analysis was carried out by AUC followed by a *t*-test. **C.** GSEA analysis in KMT2A-r AML THP1 cells treated with TRIM24 PROTAC revealed that the top affected pathways are GSKα, MYC, and BRD9 (PMID: 29507391). **D.** The mRNA expression correlation between GSK3β, TRIM24, and SPOP was investigated using DepMap leukemia cell lines (https://depmap.org/portal/) and TCGA patient samples (https://www.cbioportal.org).

Fig. S6. *In vivo* combinatory effects of GSK3i and BETi in SEM xenografts. A. Schematic diagram for *in vivo* combination therapy assay. SEM cells expressing sgSPOP or sgNT were injected into NSG mouse recipients, followed by dosing of BETi, GSK3i, or the combination. A weekly flow cytometry assay was performed to determine the leukemia burden in mouse peripheral blood. **B-C.** Leukemia burden in mouse peripheral blood. SEM leukemia burden was measured by flow cytometry at 3 (**B**) and 5 (**C**) weeks post-injection. Five mice were included in each treatment arm. *P*-values were determined by a 2-tailed *t*-test. *P <0.05, **P <0.01, ***P <0.001; n.s., not significant.

Fig. S7. Characterization of KMT2A-r ALL in PDX cells. A. Fluorescence in situ hybridization (FISH) was performed to validate the presence of the KMT2A–AFF1 fusion in 3 ALL PDX cells (PDX1-3)**. B-C.** Ex vivo sensitivity of PDX cells to BETi (ABBV-744, **B**) and GSK3i (CHIR-98014, **C**). ABBV-744 sensitivity was determined by high-content imaging assay. **D.** BETi (ABBV-744) and GSK3i (CHIR-98014) effects in PDX1 cells isolated from mice that relapsed after BETi/GSK3i combination.
