# Supplementary figures and images for: Interrogating bromodomain inhibitor resistance in KMT2A-rearranged leukemia through combinatorial CRISPR screens
Source: Proc Natl Acad Sci U S A. 2023 Apr 10;120(16):e2220134120. doi: 10.1073/pnas.2220134120 (PMC10120025; doi:10.1073/pnas.2220134120)

Figure S1

A

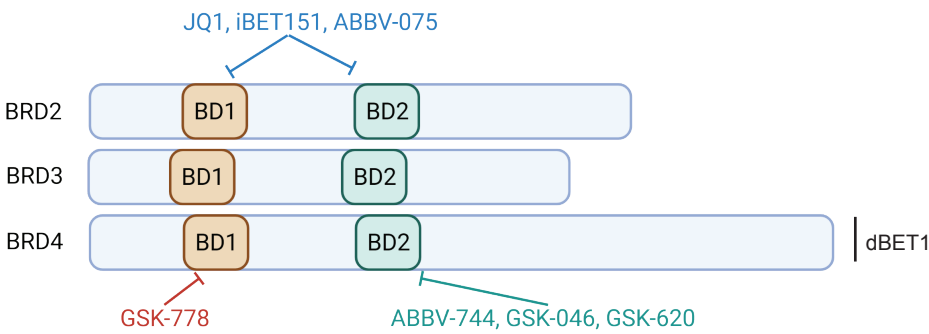

B

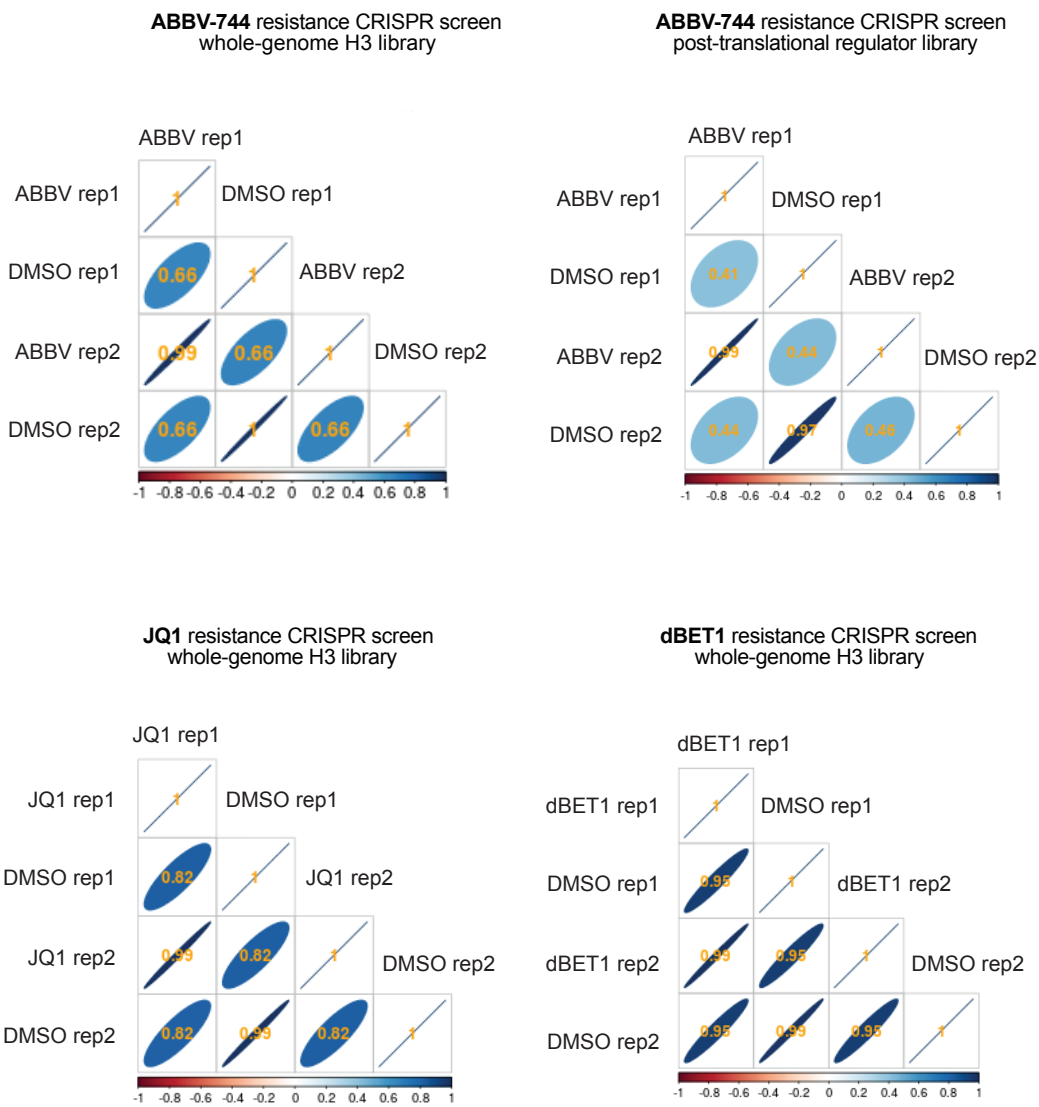

Supplement: Supplementary file 2 — Appendix 02 (PDF) [file pnas.2220134120.sapp2.pdf]

Figure S2

A

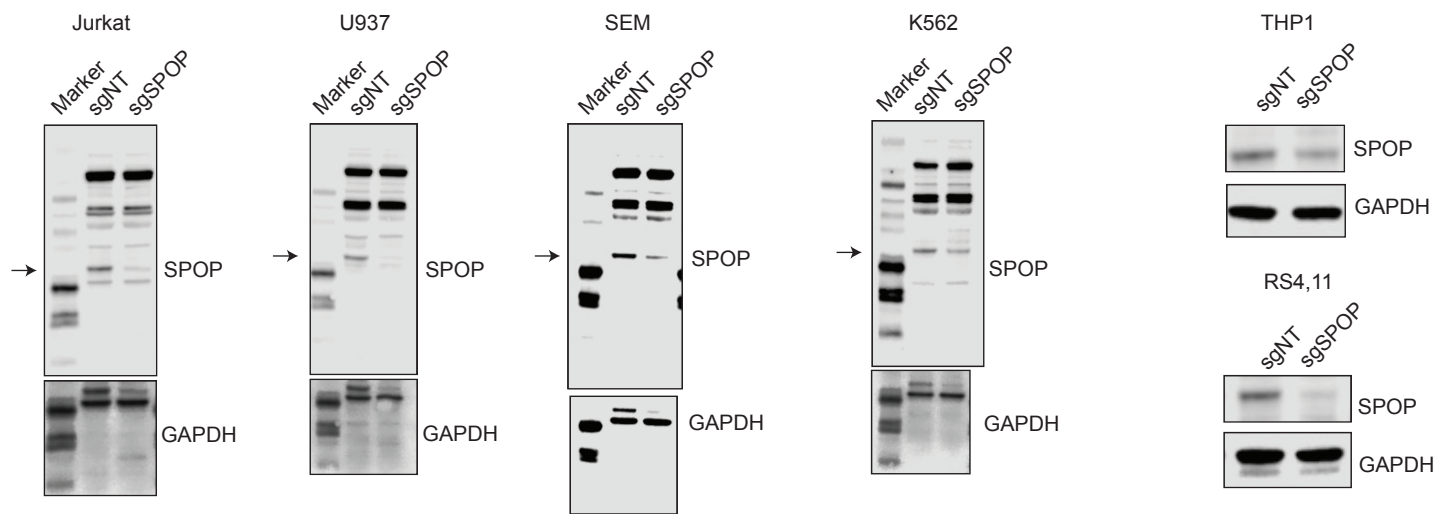

B

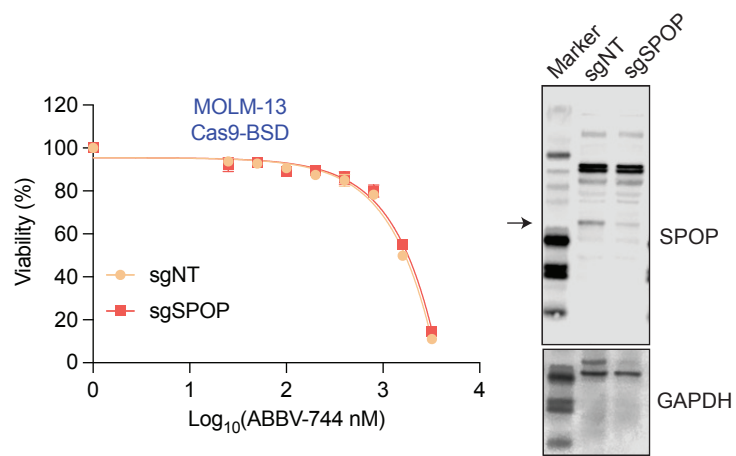

Supplement: Supplementary file 3 — Appendix 03 (PDF) [file pnas.2220134120.sapp3.pdf]

Figure S3

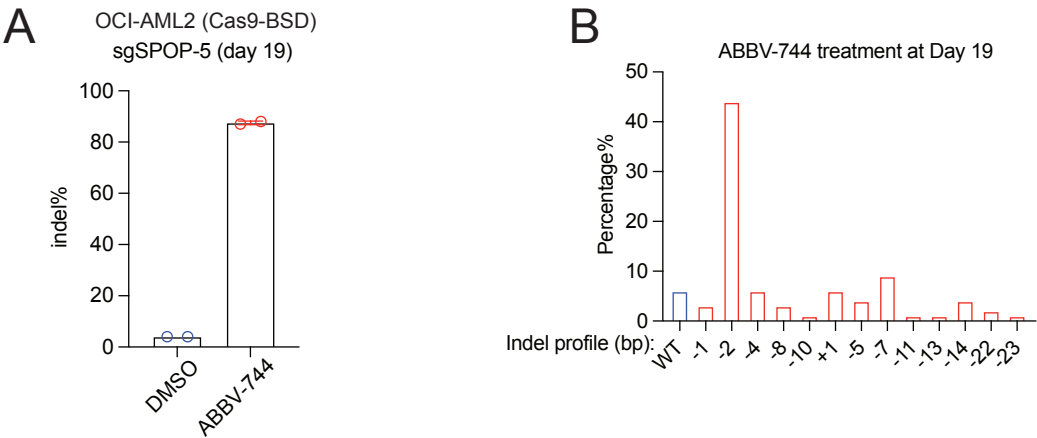

Supplement: Supplementary file 4 — Appendix 04 (PDF) [file pnas.2220134120.sapp4.pdf]

Figure S4

A

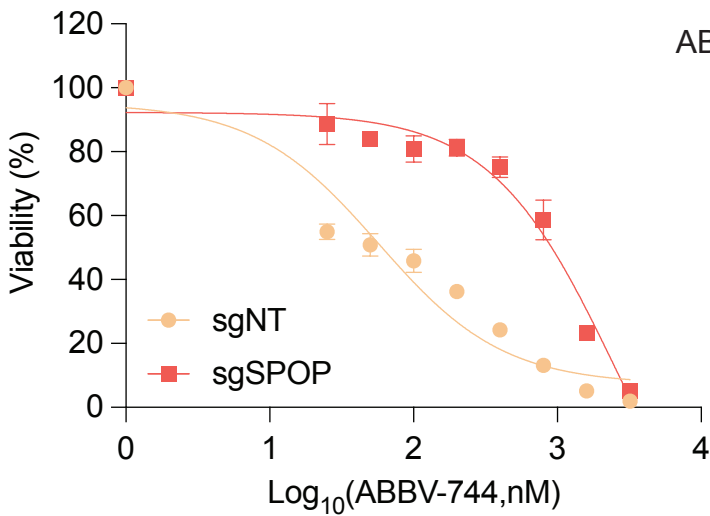

B

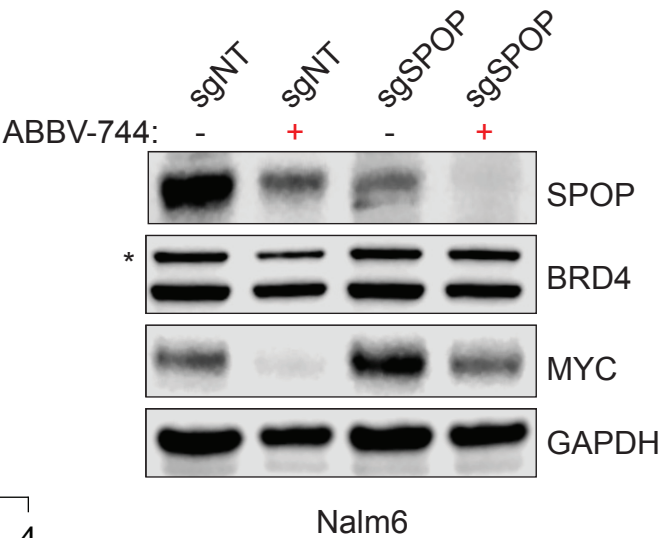

Supplement: Supplementary file 5 — Appendix 05 (PDF) [file pnas.2220134120.sapp5.pdf]

Figure S5

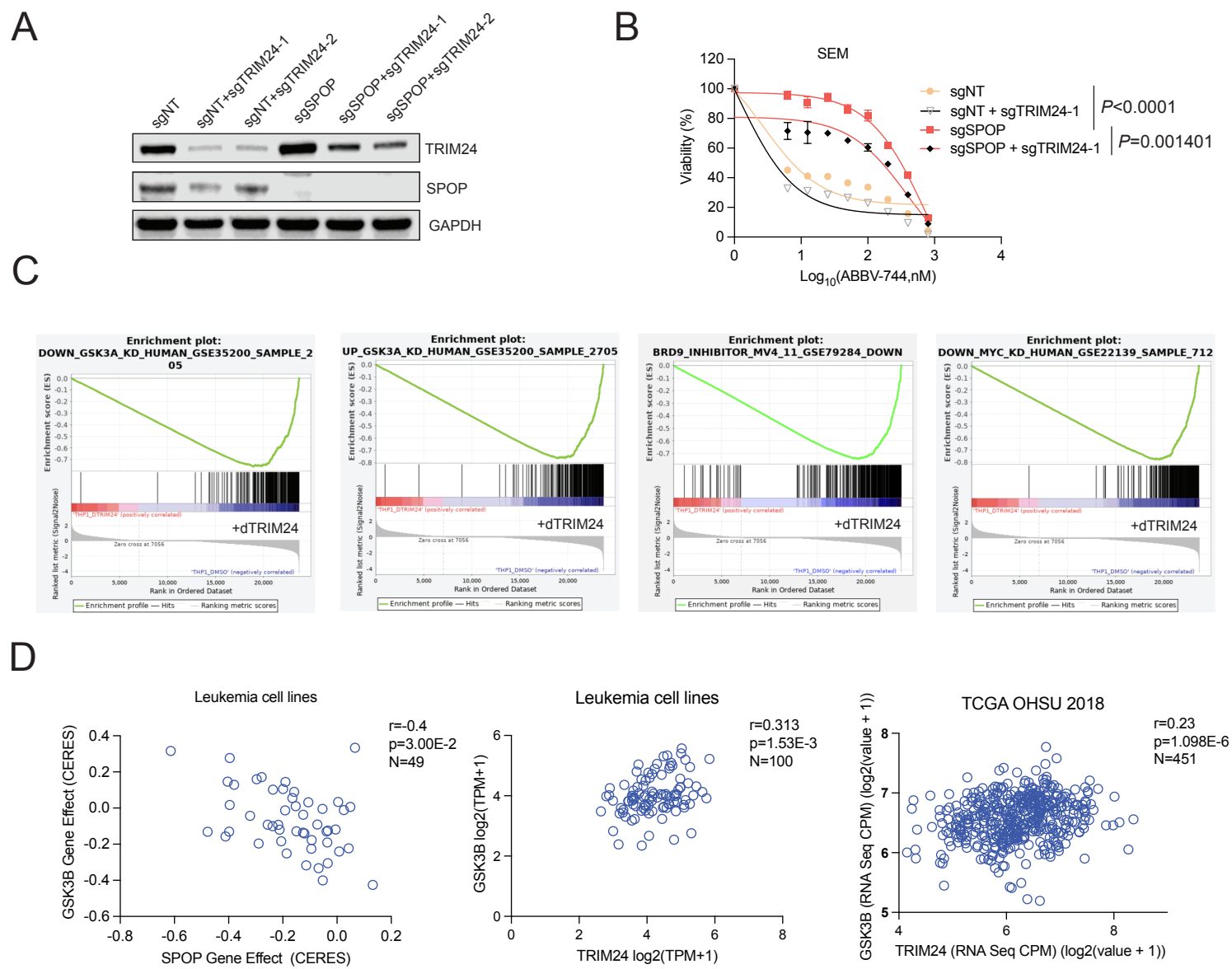

Supplement: Supplementary file 6 — Appendix 06 (PDF) [file pnas.2220134120.sapp6.pdf]

Figure S6

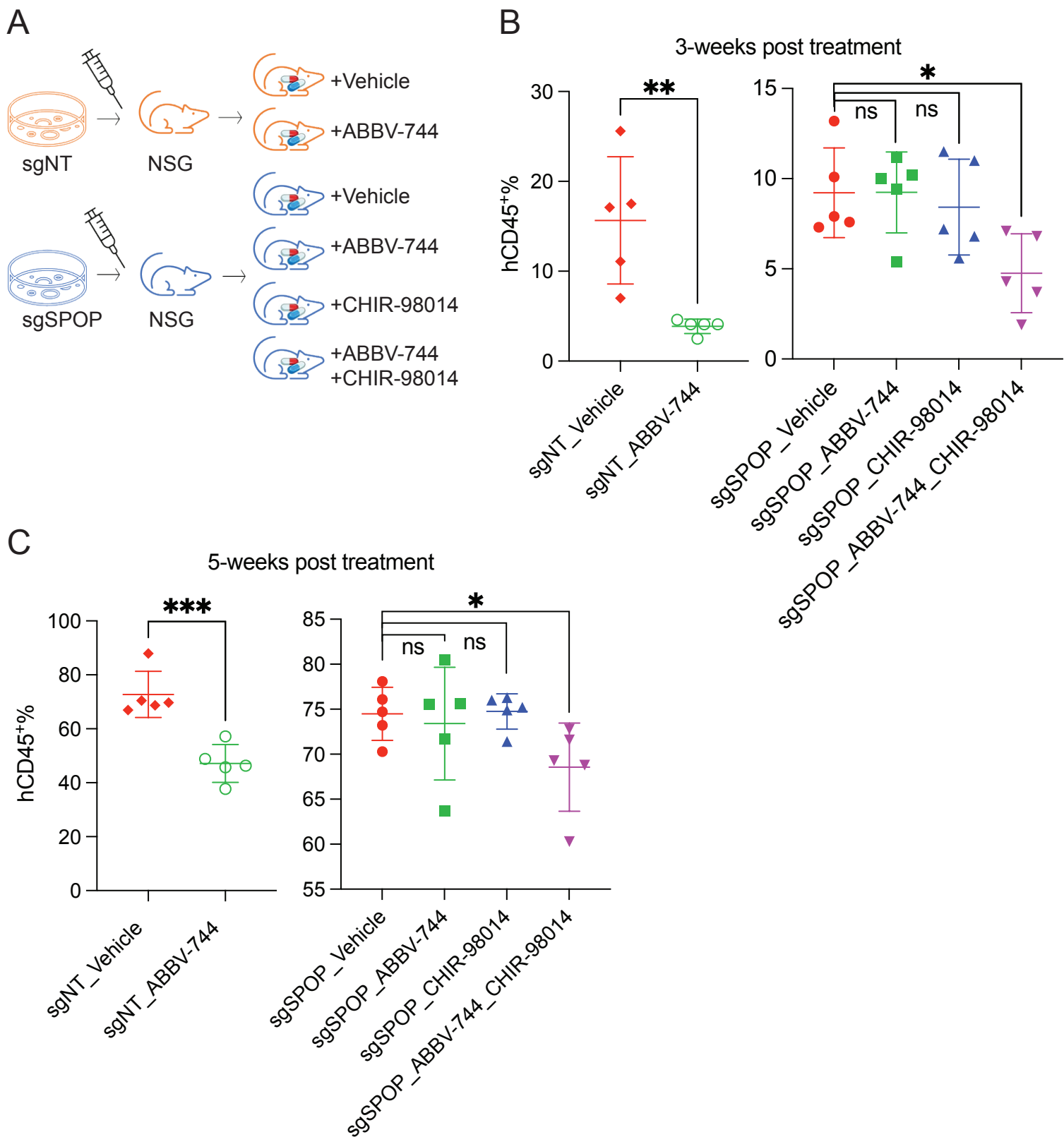

Supplement: Supplementary file 7 — Appendix 07 (PDF) [file pnas.2220134120.sapp7.pdf]

Figure S7

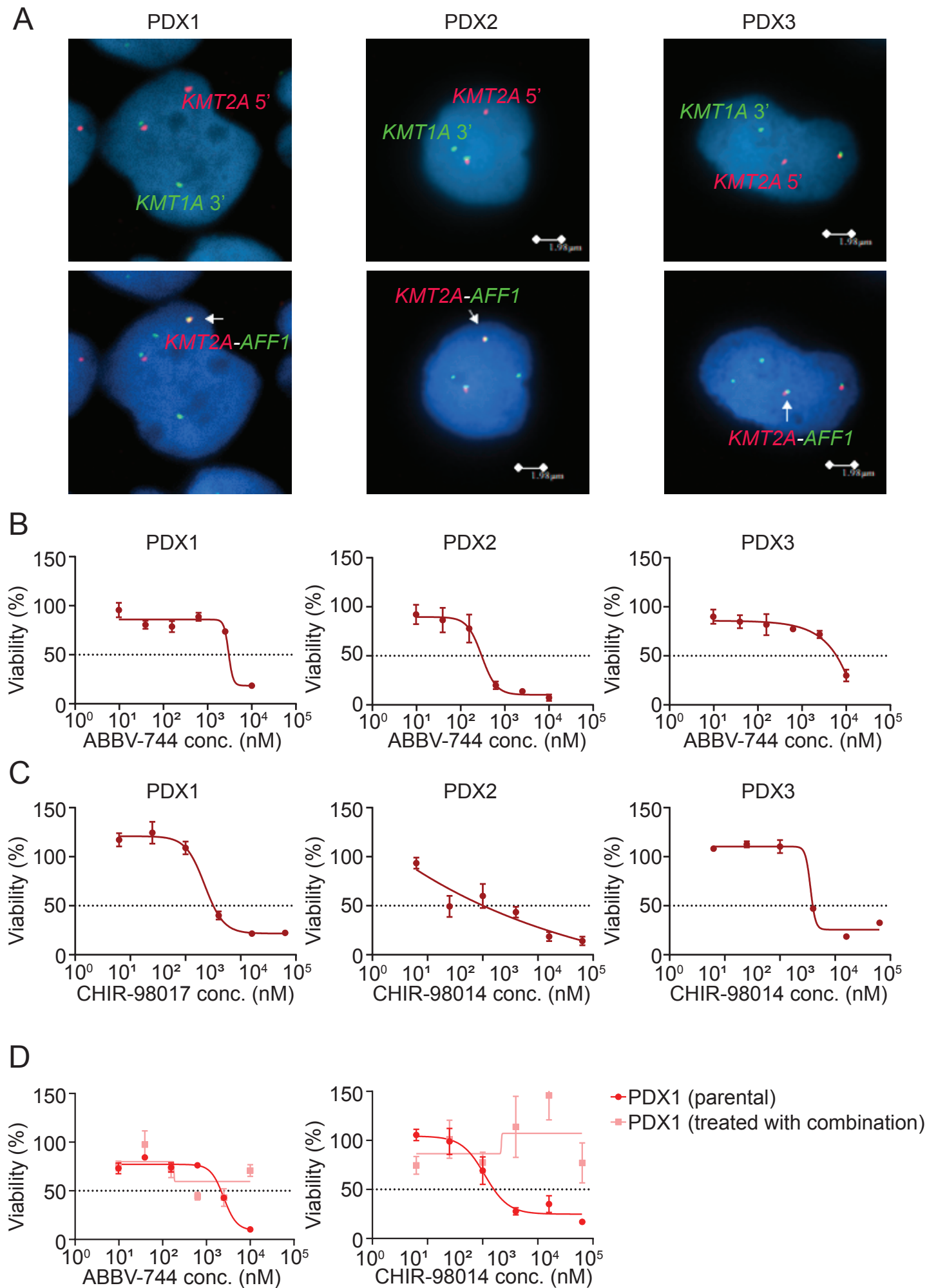

Supplement: Supplementary file 8 — Appendix 08 (PDF) [file pnas.2220134120.sapp8.pdf]
